# Tecnologias educacionais utilizadas para promoção do autocuidado de pessoas com diabetes mellitus: revisão integrativa

**DOI:** 10.1590/0034-7167-2023-0049

**Published:** 2023-12-08

**Authors:** Jennyfer Soares de Sá, Martin Dharlle Oliveira Santana, Mayara Góes dos Santos, Júnior Cesar de Souza Benedito, Elen Ferraz Teston

**Affiliations:** I Universidade Federal do Mato Grosso do Sul. Campo Grande, Mato Grosso do Sul, Brazil; II Universidade Estadual do Tocantins. Palmas, Tocantins, Brazil

**Keywords:** Diabetes Mellitus, Educational Technology, Nursing, Self Care, Health Education, Diabetes Mellitus, Tecnología Educacional, Enfermería, Autocuidado, Educación en Salud, Diabetes Mellitus, Tecnologia Educacional, Enfermagem, Autocuidado, Educação em Saúde

## Abstract

**Objective:**

To summarize the scientific productions that used educational technologies to promote self-care for people with diabetes.

**Method:**

Integrative review carried out from October 2022 to January 2023, in the databases: LILACS; Scopus; Embase; PubMed/MEDLINE and CINAHL. The search was paired, and the sample consisted of ten articles. The levels of evidence were analyzed by the Hierarchy of Evidence for Intervention Studies, and the results were synthesized for interpretation of the findings.

**Results:**

The educational technologies identified to promote self-care were: mobile applications, interactive platforms, print, telemonitoring, video and simulation. The focus of the contents was on the promotion of foot care, prevention of neuropathy, self-management, knowledge, and expectation of people with diabetes and prevention of acute complications.

**Conclusion:**

The synthesis of knowledge about educational technologies to promote self-care for people with diabetes pointed to the need for robust evidence.

## RESUMO

**Objetivo:**

Sumarizar as produções científicas que utilizaram tecnologias educacionais para promoção do autocuidado de pessoas com diabetes.

**Método:**

Revisão integrativa realizada no período de outubro de 2022 a janeiro de 2023, nas bases de dados: LILACS; Scopus; Embase; PubMed/MEDLINE e CINAHL. A busca aconteceu de forma pareada, e a amostra foi constituída por dez artigos. Os níveis de evidência foram analisados pelo *Hierarchy of Evidence for Intervention Studies,* e os resultados foram sintetizados para interpretação dos achados.

**Resultados:**

As tecnologias educacionais identificadas para promoção do autocuidado foram: aplicativos móveis, plataformas interativas, impressos, telemonitoramento, vídeo e simulação. O enfoque dos conteúdos foi na promoção do cuidado com os pés, prevenção de neuropatia, autogestão, conhecimento e expectativa de pessoas com diabetes e prevenção de complicações agudas.

**Conclusão:**

A síntese do conhecimento sobre as tecnologias educacionais para promoção do autocuidado de pessoas com diabetes apontou a necessidade de evidências robustas.

## RESUMEN

**Objetivo:**

Sintetizar las producciones científicas que utilizaron tecnologías educacionales para promoción del autocuidado de personas con diabetes.

**Método:**

Revisión integradora realizada entre octubre de 2022 hasta enero de 2023, en las bases de datos: LILACS; Scopus; Embase; PubMed/MEDLINE y CINAHL. La búsqueda ocurrió de manera pareada, y la muestra fue construida por diez artículos. Los niveles de evidencia fueron analizados por el *Hierarchy of Evidence for Intervention Studies,* y los resultados fueron sintetizados para interpretación de los hallados.

**Resultados:**

Las tecnologías educacionales identificadas para promoción del autocuidado fueron: aplicativos móviles, plataformas interactivas, impresos, telemonitorización, vídeo y simulado. El enfoque de los contenidos fue en la promoción del cuidado con los pies, prevención de neuropatia, autogestión, conocimiento y expectativa de personas con diabetes y prevención de complicaciones agudas.

**Conclusión:**

El síntesis del conocimiento sobre las tecnologías educacionales para promoción del autocuidado de personas con diabetes señaló la necesidad de evidencias robustas.

## INTRODUCTION

Diabetes mellitus (DM) is a worldwide health problem, given its magnitude. The epidemiological, social, and economic conception explains how the representation of the disease evolves in the population, especially in developing countries. It is estimated that 8.3% of the world population lives with DM, and projections indicate that the impact of the disease on the population in the coming decades will correspond to 9.6% of the more than 486 million cases predicted by 2045. In the world ranking, Brazil ranks fifth among countries, with 16.8 million people with DM and has prospects of 21.5 and 26 million in 2030 and 2045, respectively^(1)^.

It should be noted that the severity of DM and the weaknesses of the actions that favor the individual’s own commitment to their health condition and self-care contribute to the worsening of the disease and the occurrence of complications^(2)^. Therefore, to mitigate and reduce the risks of complications and expand the possibilities of care actions, considering an expanded concept of health, the World Health Organization issued, in 2019, a guideline on the use of information technologies (e.g., internet and mobile resources) as a resource to strengthen health education actions^(3)^.

It is worth noting that the traditional care model, which focuses on the drug treatment of the disease and the provision of general information by health professionals, has already proved to be expensive and innocuous in the care of people with chronic conditions. This is because the actions do not stimulate the individual’s self-care and commitment to their health condition^(4)^.

From this perspective, education actions focused on health promotion are essential to help individuals themselves to evaluate their behaviors and identify the need for changes aimed at a healthy lifestyle. This is because they provide the opportunity to build strategies based on the different needs of individuals, considering lifestyle, social contexts, living spaces and the main health problems^(5)^. In this context, the use of educational technologies (ET) as a resource for health education has shown promise^(6)^. Studies show that the applicability of ET by health professionals contributes to people with chronic conditions to obtain greater engagement in care actions for their health condition^(7, 8)^.

In view of the above, it is expected that the synthesis of the knowledge produced on the use of ET to promote self-care for people with diabetes can assist health professionals in identifying existing and possible resources to be incorporated into practice, as well as identifying gaps related to the focus of the care actions addressed by these ETs.

## OBJECTIVE

To summarize the scientific productions that used educational technologies to promote self-care for people with diabetes.

## METHODS

### Ethical Aspects

As this is a grounded study with scientific articles in the public domain, the review procedures of the research ethics committee were waived.

### Study type

Integrative literature review research^(9)^, anchored in six subsequent stages: identification of the problem; elaboration of the guiding question; literature search; data collection by structured instrument; data analysis; and presentation of the review with dissemination of the results^(10)^.

The PVO strategy was used, in which “P” is the population of interest or condition/problem investigated (diabetes mellitus); “V”, the variable of interest (educational technologies); and “O”, the outcome to be analyzed (health promotion/self-care). Based on this, the guiding question of this research was structured: What scientific productions used educational technologies to promote self-care in people with diabetes?

### Eligibility criteria, sources, and search strategies

The integrative review was conducted in Campo Grande, capital of the state of Mato Grosso do Sul, Brazil. The study was conducted from October 2022 to January 2023. The database search followed the recommendations of the Preferred Reporting Items for Systematic Reviews and Meta-Analyses (PRISMA) report^(11)^.

Relevant databases in the area of health and nursing were selected for the search of primary data, namely: Latin American and Caribbean Health Sciences Literature (LILACS); Scopus (Elsevier); Embase (Elsevier); National Library of Medicine (PubMed/MED-LINE); Cumulative Index to Nursing and Allied Health Literature (CINAHL). Access to them was through the Proxy of the Federal University of Mato Grosso do Sul, via the CAPES Journals portal.

For this purpose, the following controlled descriptors of the Medical Subject Headings (MeSH) were used: “diabetes mellitus”, “Educational Technology”, “self-care”. In order to perform the crossings between them, the boolean operators AND and OR were used (Chart 1).

**Chart 1:** Description of search strategies and number of articles retrieved in each database, 2022

Databases	Search strategy	Results
LILACS	(“diabetes mellitus”) AND (“Educational Technology”) AND (“self-care”)	15
CINAHL	MH Exact Subject Heading (MH “Diabetes Mellitus”) AND MH Exact Subject Heading (MH “Educational Technology”) AND MH Exact Subject Heading (MH *“*self-care*”*) OR MH Exact Subject Heading (“Self-management”)	5,490
SCOPUS	KEY [(“Diabetes Mellitus”) AND (“Educational Technology”) AND (“Self-care”)]	86
EMBASE	‘diabetes mellitus’/exp AND ‘educational technology’/exp AND ‘self-care’	12
PUBMED/MEDLINE	((“diabetes mellitus”[MeSH Terms]) AND (“Educational Technology”[MeSH Terms])) AND (“self-care”[MeSH Terms])	15

Primary studies published in the last ten years (2012 to 2022), in English, Portuguese or Spanish, that addressed educational health technologies aimed at people with diabetes to promote self-care were considered eligible. The time frame is justified by the volume of recovered articles not directed to the objective of the review and the need to apprehend more recent studies. Duplicate articles, letters to the editor, literature reviews, conference abstracts and book chapters were excluded.

The searches were carried out by two researchers, independently and simultaneously, in August 2022, in which the same result was found in the five selected databases. The publications were stored and organized through a reference manager (Zotero), which enabled the identification and exclusion of duplicate articles.

The articles retrieved and pre-selected in the virtual libraries were screened by the main researcher according to the guiding question of the study, through the reading of titles and abstracts, and the divergences were analyzed by a second researcher. Then, the selected articles were read in full to extract the data and variables of interest, in a structured spreadsheet in Microsoft Excel, according to the Critical Appraisal of the Evidence: Part I^(12)^.

### Data analysis

Levels of evidence were analyzed based on the Hierarchy of Evidence for Intervention Studies, which classifies studies into: Level I – Systematic review of meta-analyses; Level II – Randomized controlled trials; Level III – Controlled trials without randomization; Level IV – Case-control or cohort study; Level V – Systematic review of qualitative or descriptive studies; Level VI – Qualitative or descriptive study; Level VII – Expert opinion or consensus^(12)^.

The results were synthesized and grouped for evaluation and interpretation of the findings, with descriptive presentation in synoptic tables.

## RESULTS

**Figure 1:**
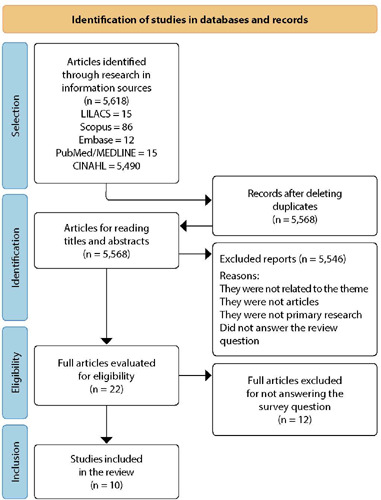
Flowchart of the search and screening process of articles based on PRISMA, 2022

Figure 1 shows the flowchart of the selection process of the primary studies included in this integrative review. The initial search in the databases identified 5,618 articles, of which 22 were selected for full reading, following the eligibility criteria. After applying the inclusion and exclusion criteria, ten primary studies were considered eligible and included in the final sample.

The articles that made up the final sample were published in the last five years, being six national articles. Regarding the type of study, there were five methodological studies, two randomized controlled clinical trials, two design studies in the area of informatics and one descriptive study. Chart 2 shows the synthesis of the studies included to compose the final sample of this integrative review (author, year of publication, type of study, sample, methodological framework, and objective).

**Chart 2:** Description of articles included in the literature review (N = 10), 2022

Author	Year of publication/Language/Country	Type of study	Number of participants	Objective
Marques^(13)^	2021/Portuguese/Brazil	Methodological	39 judges (29 in nursing, 10 with professional experience in information and communication technology) and 15 people with type 2 diabetes mellitus	To describe the process of validation of a multimedia application on a mobile platform to promote foot care for people with diabetes.
Menezes^(14)^	2022/Portuguese/Brazil	Methodological	31 nurses, 6 technicians and 15 people with diabetes mellitus	To describe the production and validation of short film educational technology for the prevention of diabetic foot ulcers.
Souza^(15)^	2021/Portuguese/Brazil	Methodological	Seven judges: two judges (technical validation) and five nurses (content validation)	To describe the construction process and evaluate the evidence of content validity and serial album appearance to prevent foot complications in diabetics.
Galdino^(16)^	2019/Portuguese/Brazil	Methodological	23 judges for internal validation and 30 people with type 2 diabetes mellitus	To describe the validation of a primer on foot self-care for people with diabetes.
Amdie^(17)^	2022/English/Ethiopia	Randomized controlled trial	40 participants: 20 received the virtual simulation-based educational program, and 20 continued with their usual diabetes care.	To evaluate the feasibility of a virtual simulation-based Diabetes Foot Care Education (DFCE) program among adult patients with diabetes in Ethiopia.
Jafari^(18)^	2021/English/Iran	Qualitative	33 participants involved in the research	To design a prototype portal based on two care models and a contextualized education program to support the self-management of patients with diabetes.
Thuah^(19)^	2019/English/Malaysia	Design and Gamification	20 participants accepted its usability.	Develop a diabetes self-management application using the Rapid Application Development (RAD) methodology.
Vluggen^(20)^	2021/English/Netherlands	Randomized controlled trial	234 gained access to the eHealth program.	To examine the efficacy of the eHealth program in a randomized controlled study.
Duarte^(21)^	2020/Portuguese/Brazil	Action research with qualitative approach	14 participants with type 2 diabetes mellitus	To analyze participants’ knowledge and expectations regarding diabetes and their health.
Silva^(22)^	2020/Portuguese/Brazil	Methodological	There were no participants	To build an application on the prevention of acute complications of diabetes mellitus, for health education of people with the disease.

In turn, Chart 3 shows the synthesis of the studies included to compose the final sample of this integrative review (intervention; technology developed; main results and conclusions; and level of evidence).

**Chart 3:** Summary of the studies included to compose the final sample of this integrative review, 2022

Reference	Intervention /Educational technology developed	Key findings and conclusions	Level of evidence
Marques^(13)^	Not performed /Application: Padcare	Application with information and language able to promote and learning and feet care	VII
Menezes^(14)^	Unrealized /Short film: Pés que te quero	Valid and suitable resource to be used in educational actions favorable to the promotion of self-care with the feet	VII
Souza^(15)^	Unrealized /Serial album	Material suitable for use in educational activities	VII
Galdino^(16)^	Not done / Printed booklet	Content considered relevant for transmitting knowledge and raising awareness to promote behavior change	VII
Amdie^(17)^	Intervention with diabetes education program based on virtual simulation followed by a structured interview to explore (acceptability, practicality) the potential impact of the intervention /Virtual simulation	The study explored the feasibility of innovative education programs to improve understanding of foot care education (DFCE) in a diverse socioeconomic context. Another purpose was to support the creation of the basis for the conceptualization of an effective program in diabetes education.	II
Jafari^(18)^	Interviews with target audience to evaluate the prototype / Prototype of interactive portal and educational program	This study provides guidelines for the development of an educational portal via the internet, focused on patient support in their social context.	VI
Thuah^(19)^	Performed the usability test: Rapid Application Development (RAD) with the Software Usability scale (SUS) to evaluate the application /Gamified application with self-presentation avatar	The gamification approach for diabetes is seen as a promising approach to promote self-management.	IV
Vluggen^(20)^	Periodic evaluations with the program / Randomized controlled trial	The multi-behavioral eHealth program decreased caloric intake of unhealthy snacks compared to the control group (d = 0.36; 95% CI 0.136 to 0.584; P = 0.002); and no significant changes were observed regarding adherence to physical activity (d = -0.14; 95% CI -0.388 to 0.109; P = 0.27), and oral hypoglycemic agents (d = 0.27; 95% CI -0.027 to 0.457; P = 0.08).	II
Duarte^(21)^	Map-speaker (MF), telemonitoring and interview / Telemonitoring	Effective in stimulating and enhancing health care	VI
Silva^(22)^	Unrealized /Interactive digital app	It is an interactive support for health education actions and an incentive for preventive self-care.	VII

The focus of the contents addressed in the different ETs was the promotion of self-care to minimize diabetes complications, especially those related to neuropathic lesions in the feet of people with DM. This content was included in five of the articles^(13, 14, 15, 16, 17)^, four of which were national publications and derived from methodological studies. In addition, among the four international studies, three addressed themes and content on self-care aimed at changing behavior and adherence to drug treatment, healthy eating, and physical activity^(18, 19, 20)^.

## DISCUSSION

The rapid evolution of ETs promotes transformations in the ways of understanding content and facilitates the building of knowledge, so that they become active educational instruments^(23)^. They can be integrated into health education and enable the optimization of learning outcomes^(24)^. In the context of nursing care, they can enhance and qualify care actions, since they help in the process of learning and self-knowledge of the individual regarding their health condition^(25)^. In the studies identified in this review, the TEs used were: printed technologies^(15, 16)^; interactive technologies and mobile applications^(18, 19, 20, 22)^; telemonitoring^(21)^; simulation^(17)^; and audiovisual production^(14)^.

According to the results of a study carried out in Iran, diabetes self-management education requires the use of tools that facilitate knowledge and contribute to the development of the skills and capacity necessary for self-care^(7)^. In addition, ETs can help establish new behaviors and skills as well as enhance preexisting skills in relation to DM. The explanation lies in the fact that, in addition to allowing an intrinsic relationship between teaching and learning, they promote the applicability of new self-care standards^(5)^.

The use of interactive platforms to build care tools was identified in most of the selected studies^(18, 19, 22)^. Along with the importance of interactivity for care, a systematic review highlighted that access to interactive technologies by people with DM has generated positive impacts on changes in habits and lifestyle. The authors evaluated the effectiveness of avatar-based gamification applied to health education; and highlighted favors regarding self-management, self-efficacy, behavior change and improved knowledge about related diseases^(26)^. The benefits can be explained through the challenges contained in the games, which activate the dopaminergic system and, consequently, benefit players^(27)^ by increasing the motivation and involvement of users with certain information^(28)^.

In this regard, it should be noted that around 29.4% of internet users use applications aimed at monitoring health, daily practice of physical exercise and nutrition. Among these applications, gamification with the interaction process as a reward has become similar to interactive games^(28)^.

An intervention study carried out in Iran showed the positive effect of self-care education through the weblog in improving the quality of life, Body Mass Index (BMI) and blood pressure of people with DM, due to the increased awareness of these people about the risks as well as about the forms of control and treatment^(7)^.

Printed materials such as booklets^(16)^ and serial album^(15)^ were also ETs identified in the analyzed studies. Regarding the use of these technologies, the authors highlighted the relevance of printed material for the context of chronic diseases^(29)^ and sensitization of individuals to self-responsibility and healthy lifestyle^(30)^.

In the scope of audiovisual production, an article^(14)^ used this ET. It should be noted that videos are an essential element for communication, storage, and transfer of information, use, production, and mass sharing^(24)^. In this context, nurses can develop validated technologies such as educational videos to promote playful educational practices^(31)^, adaptable to different scenarios and facilitating the involvement of individuals in treatment^(16)^.

In turn, the applications were recurring technologies, in this review, as tools to deal with the problem at hand. Similarly, a study carried out in Australia, which analyzed the experiences of people with chronic diseases in the self-management process, pointed out that mobile applications have great visual representation, encourage self-motivation and, sometimes, have an easy-to-use design. They also facilitate health education actions aimed at monitoring glucose, food, and other self-care actions. In addition, health professionals who discuss care themes through apps encourage self-reflection on diabetes control^(32)^. However, a review study showed that there is a reduced number of applications that allow the interaction between the person with DM and health professionals in monitoring the self-management of the health condition^(33)^.

In addition, it is important to highlight that five of the studies from this review focused on health prevention and reduction of microvascular and macrovascular complications caused by DM, especially foot ulcers due to peripheral neuropathy. In this context, it is important to note that people with diabetes who have developed foot ulcers have a high risk of amputation and a considerable reduction in life expectation^(34)^. For this reason, the implementation of preventive actions, interdisciplinary monitoring, and health education activities can minimize this complication by 44% to 85% and thus reduce the risk of amputation^(35)^. Both national and international literature indicate that low socioeconomic status and low education are predisposing factors for amputations^(36, 37)^.

Thus, several technological tools have been used to improve engagement and participation in learning sessions. In this context, collaborative and continuous efforts are necessary to identify or create the appropriate ET for an efficient education^(24)^. In this regard, the different uses of educational technologies for people with DM are shared and integrated to facilitate learning, so that the adherence of these individuals can interrupt the fragilities of knowledge in relation to the essence of self-care^(6)^.

However, it is necessary to emphasize that only the international studies^(18, 19, 20)^ of this sample emphasized behavior change and other themes necessary to promote self-care, such as: physical exercise, healthy eating, glycemic control and professional support. In this sense, although scientific productions on DM are frequent in the national context, it was observed that the focus is on preventing complications of the disease. Therefore, considering the complexity of care for a chronic condition and the expanded concept of health, with a view to comprehensive care, it is necessary to develop educational actions and resources with emphasis on health promotion^(38)^.

### Study limitations

The limitation of this study was the publication period, which may have restricted the reach of a greater number of articles.

### Contributions to the area

Among the main potentialities of this review, we highlight the gap identified regarding the use of educational technologies focused on behavior change, an essential condition to be encouraged to promote self-care, especially by the Family Health Strategy team for people with chronic conditions.

## CONCLUSION

The educational technologies to promote self-care in people with diabetes identified in this study were: printed technologies, interactive technologies, mobile applications, telemonitoring, simulation and audiovisual production. It was found that there is a need for robust evidence on educational technologies to promote self-care for people with diabetes.
