# ERRATUM

**DOI:** 10.1590/0034-7167.20247702e05

**Published:** 2024-06-14

In the article “Educational technologies used to promote self-care for people with diabetes mellitus: integrative review”, with DOI number: https://doi.org/10.1590/0034-7167-2023-0049, published in Revista Brasileira de Enfermagem, 2023;76(Suppl 4):e20230049, in the title:

Where it read:

**Tecnologias educacionais utilizadas para promoção do autocuidado de pessoas com diabetes *mellitus*: revisão integrativa**

*Tecnologias educacionais utilizadas para promoção do autocuidado de pessoas com diabetes mellitus: revisão integrativa*

*Tecnologías educacionales utilizadas para la promoción del autocuidado de las personas con diabetes mellitus: Revisión Integradora*

It reads:

**Educational technologies used to promote self-care for people with diabetes mellitus: integrative review**

*Tecnologias educacionais utilizadas para promoção do autocuidado de pessoas com diabetes mellitus: revisão integrativa*

*Tecnologías educacionales utilizadas para la promoción del autocuidado de las personas con diabetes mellitus: Revisión Integradora*

