# Supplementary material for: ERRATUM
Source: Rev Bras Enferm. 2024 Jun 14;77(2):e2024n2e05. doi: 10.1590/0034-7167.20247702e05 (PMC11178313; doi:10.1590/0034-7167.20247702e05)
Supplement: Related texts

# Correction: ERRATA

**DOI:** 10.1590/0034-7167.20247702e05pt

No artigo “Tecnologias educacionais utilizadas para promoção do autocuidado de pessoas com diabetes mellitus: revisão integrativa”, com número DOI: https:// doi.org/10.1590/0034-7167-2023-0049pt, publicado no periódico Revista Brasileira de Enfermagem, 2023;76(Suppl 4):e20230049, no título:

Onde se lia:

**Tecnologias educacionais utilizadas para promoção do autocuidado de pessoas com diabetes *mellitus*: revisão integrativa**

*Tecnologias educacionais utilizadas para promoção do autocuidado de pessoas com diabetes mellitus: revisão integrativa*

*Tecnologías educacionales utilizadas para la promoción del autocuidado de las personas con diabetes mellitus: Revisión Integradora*

Leia-se:

**Tecnologias educacionais utilizadas para promoção do autocuidado de pessoas com diabetes *mellitus*: revisão integrativa**

*Educational technologies used to promote self-care for people with diabetes mellitus: integrative review*

*Tecnologías educacionales utilizadas para la promoción del autocuidado de las personas con mellitus: Revisión Integradora*
